# Participant and GP perspectives and experiences of screening for undiagnosed type 2 diabetes in community pharmacy during the Pharmacy Diabetes Screening Trial

**DOI:** 10.1186/s12913-023-10269-1

**Published:** 2023-12-01

**Authors:** Ines Krass, Michael J. Twigg, Bernadette Mitchell, Frances Wilson, Mohammadreza Mohebbi, Peta Trinder, Sophy T. F. Shih, Rob Carter, Vincent L. Versace, Kevin McNamara

**Affiliations:** 1 School of Pharmacy, University of Sydney, Sydney, NSW 2006 Australia; 2 School of Pharmacy, University of East Anglia, Norfolk, NR47TJ UK; 3 Biostatistics Unit, Faculty of Health, Deakin University, Geelong, VIC Australia; 4 Deakin Rural Health, School of Medicine, Faculty of Health, Deakin University, Warrnambool, VIC Australia; 5 Kirby Institute, Faculty of Medicine and Health, University of New South Wales, Sydney, NSW Australia; 6 Deakin Health Economics, Institute of Health Transformation, School of Health and Social Development, Faculty of Health, Deakin University, Geelong, VIC Australia

**Keywords:** Community pharmacy, Opportunistic screening, Type 2 diabetes, GP referral, Screening participant experiences, GP perspectives, Point-of-care

## Abstract

**Background:**

The Pharmacy Diabetes Screening Trial (PDST) evaluated three approaches to screening for undiagnosed type 2 diabetes mellitus (T2DM) in community pharmacy: (1) paper-based risk assessment (AUSDRISK) alone; and AUSDRISK followed by a point of care test if AUSDRISK ≥ 12; with either (2) HbA1c; or (3) small capillary blood glucose Test (scBGT). This paper reports the perspectives and experiences of the pharmacy screening service of two key stakeholder groups: screening participants and general practitioners (GPs).

**Methods:**

All referred participants (*n* = 2242) received an online survey to determine the outcome of the referral, as well as their level of satisfaction with the service. In addition, a random sample of 2,989 (20%) of non-referred participants were surveyed to determine their overall experience and level of satisfaction with the service. GPs to whom participants were referred were contacted to establish if, since the date of the screening service, their patient had (1) been to see them; (2) had further tests performed (FBG, RBG, OGTT, HbA1c); or (3) been diagnosed with diabetes or prediabetes. Descriptive statistics were reported for quantitative data. Factors associated with visiting the GP following screening were assessed using multivariable logistic regression. Qualitative data were analysed using content analysis.

**Results:**

Response rates 16% (*n* = 369) and 17% (*n* = 520) were achieved for the three-month referred and non-referred participant surveys, respectively. Over 90% of respondents were very positive about the screening service (*n* = 784/853) and would recommend it to a family member or friend (*n* = 784/853). Participants also reported making significant improvements in diet and exercise, because of the screening. Among referred respondents, those who received a POC test were twice as likely to visit their GP compared to those who received a risk assessment only (OR 2.11 95% CI 1.46–3.06**)**. GPs (15.8% response rate, *n* = 57/361) indicated that the referral worked well and that recommendations for follow-up care by the pharmacist were appropriate.

**Conclusion:**

Opportunistic screening of individuals during routine encounters with the community pharmacy in a previously undiagnosed population has been shown to foster positive engagement with consumers and GPs, which may assist in reducing the burden of T2DM on the individual and the community.

**Supplementary Information:**

The online version contains supplementary material available at 10.1186/s12913-023-10269-1.

## Introduction

In Australia, an estimated 500,000 adults have undiagnosed type 2 diabetes mellitus (T2DM) and a further 2 million are predicted to have prediabetes [1–3]. As the risk of complications for those with established diabetes can be reduced by enabling timely intervention through early case detection, screening is recommended in many national [4] and international guidelines [5]. Most screening for T2DM is delivered via general practitioners (GPs); however, approximately 15% of Australians did not visit a GP at all during 2018 (3.75 million). Social disadvantage, ethnicity, and remoteness are some of the reasons that people typically see their GP less often [6]. Low levels of GP contact are associated with sub-optimal health outcomes for patients [7] as well as providing less opportunity for screening service provision. Furthermore, even those who visit GPs regularly may experience suboptimal rates of T2DM and cardiovascular disease (CVD) risk factor detection and management, including vulnerable groups such as those with mental illness and kidney disease [8–10].

In recent decades, increasing attention has focused on the need and opportunity to enhance access to prevention in primary care through integrated care with community pharmacy [11]. Community pharmacies are freely accessible, widely available and in frequent contact with individuals who are either healthy or unwell. This provides an opportunity to engage people along the health spectrum, including hard-to-reach populations who do not utilise other health services. Australians visit a community pharmacy an average of 14 times per year, the highest usage of all the major health care providers [12]. Moreover, evidence from a systematic review suggests that community pharmacies are feasible sites for screening and that a significant number of risk factors, such as high blood pressure, cholesterol, and diabetes risk, were correctly identified in community pharmacies [13].

Pharmacy-based screening services, therefore, can enhance access to screening as well as provide a gateway to GPs and other health services using a risk assessment and POC testing in a coordinated referral screening process. This aligns directly with the Australian National Diabetes Strategy [14] that aims to coordinate existing limited health care resources across all levels of government to reduce the impact of T2DM in the community. The recently completed Pharmacy Diabetes Screening Trial (PDST) was a landmark pharmacy trial, involving 14,089 participants at 339 sites across Australia that accounted for remoteness and socio-economic conditions. It compared the effectiveness and cost-effectiveness of three feasible approaches to screening for undiagnosed T2DM or risk of T2DM. Community pharmacies were selected from geographical groups of co-located postcodes and randomly allocated to three investigation groups, viz: (1) paper-based risk assessment alone using the Australian Diabetes Risk (AUSDRISK) score [15] (Group A); (2) AUSDRISK followed by a POC HbA1c test (Group B) if AUSDRISK score was elevated; and (3) AUSDRISK followed by a POC small capillary blood glucose test (scBGT) (Group C) if AUSDRISK score was elevated [16].

The protocol for referral of screening participants to their GP was adapted from the Royal Australian College of General Practitioners (RACGP) guidelines [4] through consultation with the PDST Expert Panel, which included national diabetes experts and key stakeholder representatives. In Group A, screening participants with an AUSDRISK score ≥ 12 were referred to the GP. In Group B, screening participants with an AUSDRISK ≥ 12 also received an HbA1c POC test in the pharmacy and those with an HbA1c ≥ 39 mmol/mol (5.7%) were referred to the GP. In Group C, screening participants with an AUSDRISK ≥ 12 also received a small capillary blood glucose test (scBGT) using a POC device in the pharmacy and those with either a fasting blood glucose (FBG) of ≥ 5.5 mmol/l or a random blood glucose (RBG) of ≥ 7.0 mmol/l were referred to the GP.

During the PDST, there were 145 confirmed newly diagnosed cases of T2DM and 338 cases of newly identified prediabetes. Consistent with one of the study hypotheses, of the three approaches to screening, the risk assessment using the AUSDRISK tool followed by a POC HbA1c test for those with AUSDRISK scores of ≥ 12, showed the highest overall detection rate of T2DM (1.5% of the total screened population) compared to Groups A (0.8%) and C (0.6%) [17].

The communication and support strategies used by the PDST pharmacies were developed in conjunction with the Expert Panel to facilitate acceptance of the diabetes screening service protocol by pharmacy clients and general practice. These included the provision of standardised letters to GPs about the trial and advice to all participating pharmacies to contact and disseminate this information to their local GP practices before commencing screening recruitment. Pharmacists delivering screening were supported by bespoke screening software (PDST Guildcare™) to ensure protocol-based screening, communication and referral, and a highly standardised approach across trial arms. A proforma referral letter that included the results of the screening was generated through the PDST Guildcare™ software for all screening participants requiring referral. Pharmacists were mandated to give a copy to each individual and to supply a copy to their nominated GP practice with their consent.

Ultimately, the effectiveness of such programs depends on the level of uptake of screening and referral by consumers and for those found to be at risk, the provision of diagnostic testing, and continuity of care by GPs. The aim of this study was therefore to explore consumer and GP experiences of the screening program and to identify factors influencing decisions to act on referrals. The level of referral uptake by screening participants together with the level of response from the GP served as a measure of how well the PDST screening service fitted into primary care.

## Methods

This analysis was a secondary mixed methods study, embedded within the PDST involving GPs and screened participants in the PDST.

### Ethics approval

Ethical approvals for the consumer and general practitioner (GP) study were obtained from the Human Research Ethics Committees at the University of Sydney and at Deakin University.

### Study sample

The study sample included all consenting screening participants who had been referred to their GP (*n* = 2,242), together with a 20% random sample of non-referred screened participants (*n* = 2,989).

A random sample of GP practices to which screened participants had been referred, was also included in the analysis (*n* = 361).

### Screened participant data collection

Data on screened participant perspectives and experiences were collected at six weeks and at three months post screening, using different approaches.

At six weeks, all referred participants were contacted by the screening pharmacist to determine if they had followed up on the referral, as well as to collect information on any subsequent testing. If they had not seen a GP, the call from the pharmacist was intended to determine the reason for not having acted on the referral and to further encourage the individual to do so.

At three months post screening participant feedback was sought via a survey that was sent to those who had consented to be contacted during follow-up. Two versions of this survey were developed: (1) for referred participants and (2) for non-referred participants.

1. All referred participants received either an online or postal survey to determine the outcome of the referral, as well as their level of satisfaction with the service.
2. A random sample of 20% of non-referred participants were surveyed to determine their overall experience and level of satisfaction with the service.

In the survey sent to referred participants, the first 2 sections were as follows: *Sect. 1) Follow-up with the GP* – six questions to determine whether the participants went to the GP and if not, did they intend to go; and *Sect. 2) Referral*—three questions to explore participant and GP use of the referral form each received from the pharmacist, including whether or not the GP already had a copy.

In both surveys, participants were asked repeat AUSDRISK questions relating to diet and exercise to allow a repeated measures analysis to be conducted. In addition, they were asked a series of open-ended questions on their experiences, perceptions, and self-reported behaviour change because of receiving the pharmacy diabetes screening service. These open-ended questions were used to facilitate a deeper understanding of respondents’ experiences and opinions regarding the trial. The key difference between the surveys was that for referred participants the survey included questions about whether they had enacted the referral and the outcomes of the referral (Additional file 1).

### GP data collection

#### GP referral

The referral to the GP from the pharmacist included a screening report showing the AUSDRISK and POC results together with a fax-back form for the GP to complete and return indicating any diabetes related blood testing since the screening date, together with any diagnosis of diabetes or prediabetes.

Each GP who did not respond by fax was contacted by phone at least two times. These follow-up phone calls were carried out by project staff within six months of the screening service date to establish the following for each screening participant:

- Had they been to see their GP since the screening service date?
- Had any blood glucose (FBG, RBG, OGTT, HbA1c) testing occurred since the screening service date? and
- Had they been diagnosed with diabetes or prediabetes since the screening service date?

#### GP feedback

Towards the end of the trial, feedback from GPs who received a participant referral was sought, using a written survey including both quantitative and qualitative components (open-ended questions).

The survey instrument included a set of 14 items on a Likert scale from 1) *“Strongly agree”* to 5) “*Strongly disagree*” to measure GPs’ attitudes to the PDST service and its value for their patients (Additional file 2). Several open-ended questions also invited respondents to provide further reflections regarding pharmacy screening for undiagnosed diabetes and diabetes risk.

All surveys to GPs were distributed by fax, as this remains the preferred communication option for most GPs. Surveys were accompanied by a cover letter encouraging participation that was signed by a GP member of the PDST Expert Panel. Practice staff were asked to fax the completed survey back to the research team.

All surveys were created and managed using REDCap (Research Electronic Data Capture) [18], a recognised web-based application designed to support data capture for research studies hosted at the University of Sydney. Data were entered onto the REDCap platform either manually in response to telephone or faxed responses, or automatically via the online data capture (in response to e-mail and SMS responses).

### Data analysis

Data were transferred to the Statistical Package for the Social Sciences (SPSS) version 24 for analysis. Descriptive statistics were reported for quantitative data: mean and standard deviation for interval data, median and interquartile ranges for ordinal or skewed interval data, and number and percent for nominal data. The chi-squared test for independent proportions was used to test for differences between groups. Univariate and multivariate logistic regression were then performed to identify demographic variables associated with visiting the GP post-screening. The explanatory variables tested included gender, age group (≥ 65 years vs < 65 years), area (Major Cities, vs Inner and Outer Regional and Rural Remote/Very Remote), having received a GP referral, having received a POC test during screening, and investigation group. Missing data are reported for any variable if they exceed 5%. The variables found to be significant in the univariate analysis were entered into a multivariable model using a forward approach. A *p*-value of < 0.2 was the criterion for entry into the multivariable model to identify a parsimonious model with the lowest Akaike's information criterion score. The model fit was tested using the Hosmer–Lemeshow statistic.

Responses to the open-ended questions in both surveys were analysed using a content analysis approach [19]. Responses were read and a coding framework developed and then re-applied to the data. The number and description of the codes along with illustrative quotes are presented.

## Results

Between 1^st^ April and 21^st^ November 2017, 2,242 surveys were sent (via email, SMS or post) to referred participants and 369 responses were received (response rate 16%). During the same period, 2,989 surveys were emailed to non-referred participants and 520 responses were received (response rate 17%). Overall, survey responses were similar between the referred and non-referred participants and demonstrated an even spread of responses from the three investigation groups, by gender, by age, and by highest screening service component provided (i.e., risk assessment plus a POC test plus referral). Table 1 describes the demographics of the surveyed sample and compares it to the full trial population. The demographic profile of the survey sample closely matched that of the full trial population.

**Table 1:** Demographics

Sample characteristic (*N* = 1365 unless stated)		Measure	Main trial total	Consumer survey total	Referred to GP (*N* = 535 (39.2%))	Not referred to GP (*N* = 830 (60.8%))
**Investigation group**	Group A (risk assessment (RA) only)	N (%)	3957 (28.0)	473 (34.7)	324 (60.6)	149 (18.0)
	Group B (RA + HbA1c)	N (%)	5165 (36.6)	471 (34.5)	169 (31.6)	302 (36.4)
	Group C (RA + capillary blood glucose)	N (%)	4971 (35.3)	421 (30.8)	42 (7.9)	379 (45.7)
**Gender**	Male	N (%)	6288 (44.5)	566 (41.5)	243 (45.4)	323 (38.9)
	Female	N (%)	7810 (55.5)	799 (58.5)	292 (54.6)	507 (61.1)
**Age group**	35 to 44	N (%)	3239 (23.1)	208 (15.2)	38 (7.1)	170 (20.5)
	45 to 54	N (%)	3608 (25.7)	287 (21.0)	79 (14.8)	208 (25.1)
	55 to 64	N (%)	3721 (26.5)	424 (31.1)	190 (35.5)	234 (28.2)
	65 to 74	N (%)	3470 (24.7)	446 (32.7)	228 (42.6)	218 (26.3)
**Pharmacy location**	Rural	N (%)	421 (3.0)	50 (3.7)	12 (2.2)	38 (4.6)
	Regional	N (%)	4773 (34.0)	481 (35.2)	191 (35.7)	290 (34.9)
	Metro	N (%)	8844 (63.0)	834 (61.1)	332 (62.1)	502 (60.5)
**Highest screening service component provided**	Initial screening	N (%)	6397 (45.6)	452 (33.1)	0	452 (54.5)
	POC test	N (%)	4582 (32.6)	378 (27.7)	0	378 (45.5)
	Referral	N (%)	3059 (21.8)	535 (39.2)	535 (100)	0
**AUSDRISK Score**^**a**^		Mean (SD)		12.6 (5.3)	17.1 (3.7)	11.7 (5.2)
**HbA1C Percentage**^**b**^** (*N* = 325)#**		Median (IQR)		5.5 (5.3–5.7)	5.8 (5.7 – 6.0)	5.3 (5.1 – 5.5)
**Capillary blood glucose (mmol/L)**^**b,c**^** (*N* = 264)**		Median (IQR)		5.5 (5.1 – 6.3)	7.9 (7.0 – 8.8)	5.4 (5.0 – 6.0)

^#^ Study was conducted at a time when HbA1c was measured as a percentage rather than mmol/mol. For reference 5.5% = 37 mmol/mol”

^a^Normally distributed

^b^Skewed data

^c^Only 16/264 were fasting samples

Based on screening participants’ self-reports obtained at the six-week and three-month follow-ups, an average of one in two had followed up with their GP; with uptake significantly higher for Group C (65%) than for Group B (56%) or Group A (44%) (p < 0.001).

### Screened participant satisfaction with the diabetes screening service

The screening service performed well on all satisfaction ratings, with more than 90% of respondents rating the service as professional or very professional (Table 2). Responses from the open-ended questions were grouped into four themes relating to (1) views on the community pharmacy, (2) feedback on the screening service, (3) changes made because of the service, and (4) the role of the GP in screening. Detailed explanation of themes and illustrative quotes are presented in Table 3.

**Table 2:** PDST participant opinions by referred status and Investigation group

**Satisfaction question** ***N* = 1365**		**Referred participants**	**Non-referred participants**	**Group A** **Risk assessment only**	**Group B** Risk assessment Plus HbA1c	**Group C** Risk assessment Plus scBGT	**Total** **(median (IQR))**
**How did you feel about the way your pharmacist explained your screening result?** (N (%) satisfied/very satisfied)		476 (89.0)	792 (95.4)	418 (88.4)	441 (93.6)	409 (97.1)	1.0 (1.0 – 2.0)^a^
**How would you describe the pharmacy diabetes screening service?** (N (%) professional/very professional)		470 (87.9)	764 (92.0)	402 (85.0)	432 (91.7)	400 (95.0)	1.0 (1.0 – 2.0)^b^
**Generally, how do you feel about the pharmacy diabetes screening service?** (N (%) satisfied/very satisfied)		453 (84.7)	756 (91.1)	387 (81.8)	424 (90.0)	398 (94.5)	1.0 (1.0 – 2.0)^a^
**What is your opinion about the diabetes screening service being available in your pharmacy in the future?** (N (%) support/strongly support)		465 (86.9)	781 (94.1)	405 (85.6)	437 (92.8)	404 (96.0)	1.0 (1.0 – 2.0)^c^
**Would you recommend the pharmacy diabetes screening service to a friend or family member?**	Yes N (%)	454 (89.2)	784 (93.6)	391 (86.1)	431 (93.7)	412 (96.3)	1238 (91.9)

^a^1: very satisfied to 5: very dissatisfied

^b^1: very professional to 5: very unprofessional

^c^1: strongly support to 5: strongly do not support

**Table 3:** Screened participant comments from the open-ended question on the participant surveys (quotes indicate investigation group and referred/not referred)

Theme	Sub-theme	Number of comments (*N* = 344 coded comments) N (%)	Example quotes
**Views on the community pharmacy**	**Accessibility**	20 (5.8)	No appointment needed it appeared…which worked for me but may be difficult for others when approached to take test when they were dropping off or picking up prescriptions. **(B, non-referral)** Might be more convenient for some people rather than making an appointment at the doctors. **(C, non-referral)**
	**Professional**	19 (5.5)	The pharmacist is so friendly and helpful. I was a bit anxious at the start, but he reassured me that I was going to be okay. He is very professional and resourceful. **(B, non-referral)**
	**Advertise**	9 (2.6)	This service should be more widely advertised, I think it's an excellent idea and very convenient **(B, non-referral)**
	**Widen screening to more people**	7 (2.0)	Screening should not be just for people who look like they might need screening. Everyone needs to be screened or engaged to discuss healthy lifestyle, as a preventative course of action in the longer term. **(A, non-referral)**
**Feedback on Sthe service**	**Supportive of the service**	66 (19.2)	I think chemists are very important in the community as they can hold all the threads together. I have not been to see a GP as I do not have one, I like/trust. A lot of doctors here in XXX have their books closed. **(A, referral)** A good service for those who never see a GP for a check-up. **(C, non-referral)**
	**Useful service**	75 (21.8)	It is a good educational tool and motivational tool. Having someone tell you out loud that you are at risk of diabetes, and it would be helpful to lose weight is motivational. For example, I have been thinking about losing weight for a long time, and little bit of extra push is always helpful. **(A, referral)** It was a good kick up the bum to how my health is going. **(C, non-referral)** This is a very good, easy to access, especially for Seniors. We don't want to negotiate buses or trams to get it done. It gives you lots of information one wouldn't usually think about, right in your daily routine. **(A, non-referral)**
	**Lack of an actual test**	16 (4.7)	I think it was a waste of time to ask me questions about my health and family history and then not give me a test because I was unlikely to need it. It was no better than a magazine or Facebook quiz. It fell well short of what I consider 'screening'. **(C, non-referral)**
	**Not satisfied**	13 (3.8)	Pharmacists have not been trained and are therefore unqualified to practice medicine in this way. Dangerous. **(C, non-referral)**
	**No need for the service**	11 (3.2)	This test was not something I feel I really needed as I am a pretty healthy, active person **(B, non-referral)**
	**Waste of money/resources**	4 (1.2)	I did it because I was in a small pharmacy waiting for scripts and would have felt obvious and rude if I didn't participate. I learned nothing and felt it was a total waste of time (though no criticism for how it was handled by the pharmacy staff, who were perfectly pleasant). Perhaps the screen is useful for high-risk ppl, but I am not! I came up with a score of zero, and I could have told you that before it started. Total waste of resources. **(B, non-referral)**
**Changes made as a result of the service**	**No changes needed**	56 (16.3)	Disturbingly, the pharmacist said I was the only person she had seen who had produced a 'green' result. Therefore, I didn't feel the need to make any changes to what I am already doing.** (A, non-referral)**
	**Made changes**	8 (2.3)	Family history so I was expecting this. I am now on insulin and tablets. **(B, referral)**
**Role of the GP in screening**		40 (11.6)	My doctor informed me it was unnecessary for me to take a Diabetes test, as he screens my blood every 6 months & keeps an eye on my health. He was very annoyed with me for having the test, as I do not have Diabetes, & he would have informed me if I did have the condition. **(A, referral)** I feel your doctor should be checking this when regular blood tests are done **(B, referral)**

### Summary of participant feedback

More than 95% of respondents (782/852) were either satisfied or very satisfied with the way the pharmacist’s professional knowledge and the way they explained their screening test results. More than 90% respondents (784/853) said that they would recommend the screening service to a family member or friend (Table 2).

*The Pharmacist was very helpful and non-judgmental......very professional. (ID: NR 297)*

*The pharmacist was very professional and knowledgeable. Great service provided; I didn't know that pharmacist can do this screen so well. Save me so much time than going to the doctor and I'm well informed. (ID: NR47)*

*My rating was low-medium risk. I already eat a healthy diet and exercise. I discussed this with the pharmacist, and she was happy with my "health program" and told me to continue! (ID: NR152)*

However, a small number of participants were not satisfied with the amount of information provided and some were disappointed that they did not receive an actual blood test in the pharmacy (i.e., either they were in Group A or their risk score did not necessitate a blood test). Approximately 10% of participants also felt that this should not be a role for the pharmacist and that the GP should be the one responsible for screening. These participants tended to indicate that they engage regularly with the GP. Nonetheless, many of these participants thought that it may be more useful for those who do not regularly attend their GP practice.

*Got a very basic answer as to if I was at risk or not and that was about it. Would like more detail about my information/results. (ID: NR273)*

*Felt that it was a bit of a waste of time and would prefer a finger prick test. (ID: R175)*

*I expected more than a questionnaire. (ID: NR8)*

#### Self-reported impact of the diabetes screening service on health behaviour

Among both referred and non-referred respondents, more than 50% reported making lifestyle changes since attending the pharmacy screening service. Several respondents who had not made any changes expressed appreciation for the reinforcement of their healthy lifestyle choices offered by the pharmacists during the screening appointment. Many participants highlighted that although they had not made any additional changes to their lifestyle as a result of the service, it was useful to confirm that they were doing the right things.

*Was a wake-up call to do something to avoid getting diabetes. (ID: R369)*

*To know my lifestyle is good and that I don't need to make a dramatic change is very pleasing. (ID: R359)*

**Fig. 1:**
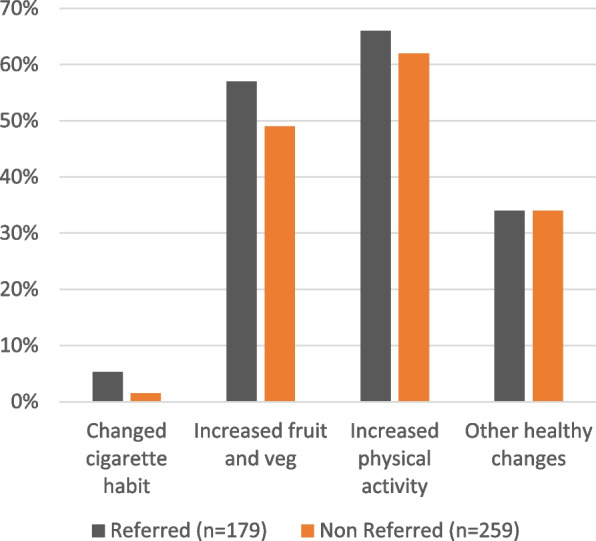
Participant survey responses – “If you made changes, which of the following lifestyle changes did you make?"

Lifestyle changes were reported across all categories but were predominantly changes to diet (increased fruit and vegetable intake) and exercise (Fig. 1).

*I have lost 14 kilos since being screened. (ID: R113)*

*Great health reminder/check-up. I have tried to eat less sugar and red meat and exercise a little more. (ID: R217)*

### Follow up of participants referred to their GP

Since only a small number (*n* = 356) of faxback forms were completed and returned by GPs, the remainder were contacted by the project team. Overall, the GP faxback form accounted for 12% of information returned to researchers, while the telephone follow-up of GPs had an 84% success rate.

### Factors influencing referral uptake by screening participants

Based on self-reports obtained at the 6-week and 3-month follow-ups with referred participants who responded to the surveys (*n* = 532), 62.1% indicated that the GP had ordered further tests. Notably, uptake of referral was significantly higher in Group B (78.6%) and Group C (76.2%) compared to Group A (64.3%) (*p* = 0.003).

In the univariate analysis, the variables tested (see data analysis) that were found to be significantly associated with visiting the GP following screening, were (1) having ‘received a GP referral’, (2) ‘having received a POC test during screening’, (3) ‘investigation group’ and (4) ‘age group’ (< 65 years vs ≥ 65 years). In the final model, ‘investigation group’ was no longer statistically significant (Table 4). In summary, those who received a referral, received a POC test, and were in age group 65 years and over, were more likely to visit their GP following the diabetes screening at community pharmacies (Table 4).

**Table 4:** Univariate and multivariable binary logistic models of factors associated with visiting the GP in the 3 months post screening (*N* = 1324)

		**Univariable**			**Multivariable**^**b**^	
**DemographicVariable**		**N (%)**	**Odds Ratio**	**95% CI**	**Odds Ratio**	**95%CI**
**Gender**	Male	281 (51.2)	1.13	(0.91–1.41)		
	Female	443 (57.2)				
**Age group (dichotomised)**	65 and over	209 (48.7)	**2.00**	**(1.58–2.52)**	**1.36**	**(1.03–1.79)**
	Under 65	515 (57.5)				
**Investigation group**	Group A (risk assessment (RA) only)^a^	231 (51.0)				
	Group B (RA + HbA1C)	263 (57.5)	**1.43**	**(1.11–1.35)**	1.27	(0.84–1.90)
	Group C (RA + capillary blood glucose)	230 (55.6)	**2.95**	**(2.21–3.93)**	0.86	(0.62–1.21)
**Pharmacy location (dichotomised)**	Rural/Regional	301 (58.6)	1.12	(0.90–1.41)		
	Metro	423 (52.2)				
**Referral**	Referred to GP	303 (60.2)	**10.20**	**(7.89–13.15)**	**14.45**	**(10.31–20.25)**
	Not referred to GP	421 (51.3)				
**Having received a POC test**	Yes	700 (56.7)	**3.61**	**(2.23–5.81)**	**2.11**	**(1.46–3.06)**
	No	24 (26.7)				

^a^Reference category

^b^Multivariable model—Hosmer Lemeshow (Goodness of fit) Chi Square 12.22; df 8; *P* = 0.14; Nagekerke R square = 0.35

### GP feedback

In total, 361 surveys were faxed to GP practices across Australia. This resulted in 57 (15.8%) responses with the majority (43, 75.4%) of those from GP practices that had engaged with the research team at some point during the trial. Responses to the Likert scale questions (Table 5) showed that while GPs gave a neutral response (neither agree or disagree) to many of the questions, they thought the referral from the pharmacy worked well and was not too time consuming or intrusive for their practice. Although GP respondents were unsure as to whether the community pharmacy was a suitable place to conduct this type of diabetes screening program, they reported that the PDST did not interfere with their relationship with the patient and that the recommendations for follow-up care made by the pharmacist to their patient were appropriate.

**Table 5:** GP responses to Likert scale questions

Item	N	Median (IQR)	Outcome	
The Pharmacy Diabetes Screening Trial was a valuable service for my patients	56	3.0 (2.0 – 4.0)	Neither agree nor disagree	
My patient(s) showed more interest in their health as a result	56	3.0 (2.0 – 3.8)	Neither agree nor disagree	
The screening referral worked well to inform me about what happened with my patients during the Pharmacy Diabetes Screening Trial	54	4.0 (2.8 – 4.0)	Agree	
The Pharmacy Diabetes Screening Trial interfered with my relationship with my patient	56	2.0 (2.0 – 3.0)	Disagree	
The Pharmacy Diabetes Screening Trial was too intrusive on my current practice	56	2.0 (2.0 – 3.0)	Disagree	
The pharmacist(s) made reasonable efforts to respond to any requests I made	55	3.0 (3.0 – 4.0)	Neither agree nor disagree	
I believe tests and measurements undertaken by pharmacist(s) were performed correctly	53	3.0 (3.0 – 4.0)	Neither agree nor disagree	
Advice provided by the pharmacist to patients after screening seemed reasonable	55	3.0 (3.0 – 4.0)	Neither agree nor disagree	
Recommendations for follow-up care made by the pharmacist(s) were appropriate	55	4.0 (3.0 – 4.0)	Agree	
The Pharmacy Diabetes Screening Trial led me to conduct extra investigations for referred patients	55	3.0 (2.0 – 4.0)	Neither agree nor disagree	
It was too time-consuming to handle referrals from pharmacists	54	2.0 (2.0 – 3.0)	Disagree	
Some of the advice provided by pharmacists conflicted with my advice	55	3.0 (2.0 – 3.0)	Neither agree nor disagree	
Overall, I trust the pharmacist(s) involved to deliver a competent diabetes screening service	55	3.0 (2.0 – 4.0)	Neither agree nor disagree	
How would you describe the community pharmacy as a place to conduct this type of diabetes screening program?^a^	52	4.0 (2.25 – 5.0)		Unsure

All responses are 1: strongly disagree to 5: strongly agree

^a^Scale: 1: Highly unsuitable to 7: Highly suitable

Responses to the open-ended questions included themes relating to the role of the community pharmacy, privacy, knowledge of pharmacists, usefulness of the service, accessibility, and duplication and fragmentation of services. Table 6 outlines these themes and provides illustrative quotes. Some GPs who were not supportive of the service felt they were already providing this screening, therefore leading to duplication. However, some also felt that it was a good service that increased awareness and could be targeted at those who didn’t regularly attend the GP practice, particularly as the community pharmacy was more accessible. The fragmentation of care was also highlighted and the need to have greater co-ordination between the GP and community pharmacy.

**Table 6:** Responses to four open-ended questions on the GP questionnaire

Theme	Number (%) of respondents^a^	Example comments
Good service/beneficial to patients	17 (33.3)	“Great pharmacist who give good advice and we work well together. Great resource for us.” **R26** “Opportunity to engage patient with health screening such as diabetes. Check if not a frequent GP attendee.” **R29**
Accessibility	7 (13.7)	“Easily accessible, have health knowledge. Blood sugar easy to check by a pharmacist.” **R29**
Not primary role of community pharmacy	12 (23.5)	“Pharmacists should focus on doing a better job of fulfilling their community pharmacy obligations before branching out into new endeavours.”** R4** “It takes away GP's primary role and gives it to the pharmacist.” **R39**
Privacy/confidentiality	6 (11.8)	“Pharmacists need private consulting rooms if they wish to participate in these sorts of activities.” **R8**
Inaccuracies/unnecessary referral	6 (11.8)	“Inaccuracy of screening as opposed to GP screening—no patients participating correctly diagnosed; they were normal on follow-up investigations. This resulted in unnecessary patient stress and blood tests (yet alone wasted time).” **R13**
Training/knowledge of pharmacists	4 (7.8)	“The chemist is having no knowledge of all patients’ medical conditions and untrained and qualified to do the test and advice patient according to results. I had patient coming scared after the test and advice from chemist.” **R15**
Duplication of care	15 (29.4)	“As stated above, it is already duplicating an existing service. Too many allied health professionals are duplicating what is already done by GPs. This is just making the cost of health care escalate.” **R44**
Lack of awareness of patient participation	7 (13.7)	“I did not receive any information about any of my patients.” **R14**
Fragmentation of care	6 (11.8)	“I communicate frequently with my community pharmacists about my patients' care and medications. I find them helpful and an important resource to optimise patient care but investigations and screening tests, I believe, is outside their remit. It is not a question of their capacity to understand or communicate the investigations but more the issue of fragmentation of care and health information. I readily accept their expertise in pharmacology and appreciate their assistance in managing patient care is this area. However, in most cases I have had experience with (possibly not in the PDST situation) screening tests offered by community pharmacists have seemed more of an opportunity to make money rather than enhancing patient care.” **R2**

^a^*N* = 51 participants responded to at least one of the open-ended questions

## Discussion

In this paper we presented the experiences and perspectives of consumers and GPs in their engagement with the pharmacy diabetes screening service implemented in the PDST, a unique clustered RCT involving a nationally representative sample of Australian pharmacies and consumers aged 35–74 years. As key stakeholders, screening participant perspectives on the appeal, value and functioning of the pharmacy screening service is key to any future national rollout. Equally critical is the issue of providing continuity of care through successful integration of a pharmacy-based diabetes screening service into primary care.

Responses to the consumer survey indicated that the screening service was well received in the pharmacy by respondents, and this was independent of their trial arm or whether they were referred to the GP. Most survey respondents felt the pharmacist explained the results well, conducted the service in a professional manner and would support the future provision of such a screening service in community pharmacy. This concurs with findings of other studies of pharmacy screening services [20–22].

While there was general satisfaction with the quality of the service, comments from the consumer surveys suggest that screening services without a finger-prick test were less valued by both consumers and pharmacists and might lead to less confidence in the result. If our findings are representative of the general view, it may help to explain increased numbers of pharmacy withdrawals and greater difficulty with participant recruitment in Group A (particularly for recruitment in rural and regional areas), where no option of finger prick testing existed. In fact, several pharmacies reported that some people demanded a POC test regardless of their AUSDRISK score and in these circumstances the pharmacists did not include them in the trial; this may have disproportionately affected Group A pharmacies. This corroborates with pharmacist feedback from previous screening trials, which showed that it may be difficult for some pharmacists to promote a paper-based service if consumers perceive it to be less comprehensive than what this pharmacy already offered [22, 23].

Referral uptake as an indirect measure of participant engagement and acceptance of the screening program also support the inclusion of a POC test as the preferred option. The provision of a referral by the pharmacist encouraged screened participants to visit their GP for follow-up thereby fostering continuity of care [24].

Another important component of the pharmacy diabetes screening service was provision of an approved information package and verbal counselling to encourage risk factor reduction by screened individuals at elevated risk. As shown in the results, a significant proportion of survey respondents had implemented a positive change in lifestyle (most likely to be increase in physical activity) to reduce their risk of T2DM. This concords with other findings in the literature demonstrating the capacity of community pharmacist interventions to encourage and realise healthy lifestyle behaviour changes in their clients [25–27].

The evaluation of GP perspectives revealed that, at least among those GPs who responded to the survey, the PDST referral from the pharmacy worked well to inform them about their referred patient’s involvement in the trial and that the recommendations for follow-up care made by the pharmacists were appropriate. They highlighted the need for such a service, especially for people who do not visit their GP regularly but raised the issue of fragmentation/duplication of care for those who were frequent attendees at their practice, as has previously been reported in the literature [28]. To avoid duplication the pharmacist needs to know what tests have already been performed for an individual client. This may be addressed in the future by sharing of information via shared electronic records [29]. Moreover, patients need to be informed of their test status by GPs. During the PDST participants were asked before enrolment if they had been tested in the past 12 months, yet many were unaware of whether they had been tested and of the results of those tests. Equally, effective communication between pharmacists and GPs will ensure that prevention messages may be reinforced for patients who are at high risk of diabetes but are unaware of it. To facilitate continuity of care and integrate the pharmacy into the primary care network, a secure effective communication channel between GPs and pharmacists must also be developed, potentially via more effective functionality and widespread adoption of shared medical records such as the MyHealth record system.

As with most studies there were limitations. Whilst every effort was made to provide standardised approaches and clear documentation, as well as appropriate answer fields in the surveys to minimise errors in responses, it is acknowledged that some data obtained from consumers and GPs was incomplete. Furthermore, as the response rates were low at 15–17%, these perspectives may not be representative of the larger cohorts of non-responders.Changes in health behaviours were also based on self-report to the pharmacist during screening or reported anonymously by the respondent in the follow-up survey, which may have been subject to bias. Moreover, it is likely that non-responders of the referred patient sample were not as engaged and therefore less likely to go to their GP or think positively about the screening as part of the follow-up survey was about whether they went to their GP for follow-up (and whether they had changed their diet and exercise). One might also infer from the low GP response rate that there was limited awareness of the screening program among many GPs to whom referrals were addressed underscoring the ineffectiveness of communication between pharmacists and GPs. However, challenges with recruiting GPs to complete surveys are well known, and lower response rates than ours for issues that GPs would certainly be aware of are commonplace; hence we should not assume the low response rate is exclusively the result of lack of awareness [30, 31]. Perceptions that screening is beyond the scope of practice of pharmacists reported by a proportion of GP respondents may also have factored into the low response rate.

Tailored strategies to improve response rate to mail surveys may be appropriate to ensure external validity of findings for future surveys of this nature, and the evidence points to the utility of various strategies. For example, inclusion of monetary incentives has been shown to increase response rates [32]. Lotteries have also been examined as a possible incentive with mixed results. Provision of scratch lotteries was found to be the most effective of this form of incentivisation [33]. A study which compared follow-up mailings and monetary incentives to maximize response rates found both to be effective. However, in the context of budgetary restraints follow-up mailings are preferred over monetary incentives. If there is limited time for survey administration, monetary incentives may be preferred over follow-up mailings [34]. In our study due to resource constraints, we were not able to deploy these types of incentives.

## Conclusion

As the prevalence of T2DM continues to rise, there is a need for novel programs that provide effective screening for undiagnosed T2DM that can reach into all geographical and socioeconomic areas of Australia – this is especially important for non-metropolitan communities where area-level SES circumstance tends to be lower [35]. The PDST addressed Goal 2 of the Australian National Diabetes Strategy [14] (i.e., to promote awareness and early detection), in that it enabled opportunistic screening of individuals during routine encounters with the community pharmacy in a previously undiagnosed population. The process evaluation provided information on some factors that influence the implementation and sustainability of the service as well as perspectives and experiences of the small proportion of consumers and GPs who responded to our surveys. Our study supports the view that to be successful a screening program must foster positive engagement among consumers, pharmacists, and GPs to reach its true potential in reducing the burden of diabetes. Better integration of pharmacists and GPs in delivery of preventive services in primary care is also needed to ensure that services are not duplicated, and that appropriate continuity of care is achieved.

### Supplementary Information

**Additional file 1.**

**Additional file 2.**

## Data Availability

The datasets generated and/or analysed during the current study are not publicly available due to Commonwealth embargo under the Sixth Community Pharmacy Trials Program funding but are available from the corresponding author on reasonable request. In terms of qualitative data it may be difficult to protect the identity of participants.
