# Supplementary material for: Participant and GP perspectives and experiences of screening for undiagnosed type 2 diabetes in community pharmacy during the Pharmacy Diabetes Screening Trial
Source: BMC Health Serv Res. 2023 Dec 1;23:1337. doi: 10.1186/s12913-023-10269-1 (PMC10693079; doi:10.1186/s12913-023-10269-1)
Supplement: Supplementary file 1 — Additional file 1. [file 12913_2023_10269_MOESM1_ESM.docx]

## Confidential

*Page 1 of 5*

Pharmacy Diabetes Screening Trial: Participant Survey (Referred)

Our records indicate that you recently participated in a Diabetes Screening Service at your pharmacy and that you were referred to your doctor for further evaluation.

We are following up on the service provided to you and on what happened afterwards. It's a short survey that will only take a few minutes to complete.

You may have already provided some of this information in a conversation with your pharmacist. However, to ensure completeness of our research data, we would appreciate you also completing this online survey.

Your answers to this survey are confidential and will not be shared with your pharmacy or your doctor.

### SECTION 1: Follow-up with Doctor

- 1. Did you follow-up with a doctor after your Pharmacy Diabetes Screening Trial appointment?

... Do you plan to follow-up with a doctor?

... Why don't you plan to follow-up with a doctor?

Yes No

(If No, please complete Section 1 then proceed to Section 5)

Yes I plan to follow-up and have made an appointment with a doctor

Yes I plan to follow-up but have not yet made an appointment with a doctor

No I do not plan to follow-up with a doctor

The pharmacy test result did not concern me enough to act on the pharmacists advice

I am too busy to follow-up

I am afraid of getting a diagnosis of diabetes The pharmacist did not advise me to follow-up Other

... Can you provide more detail on why you do not plan to follow-up with a doctor?

- 1. Which doctor did you see? (name)
  2. Which STATE or TERRITORY is the doctor located in? NSW

VIC QLD SA WA NT ACT TAS

- 1. Which SUBURB is the doctor located in?
  2. Please provide the doctor's STREET ADDRESS (if known)
  3. Please provide the doctor's PHONE NUMBER (no spaces, if landline include area code)

# Appendix 20: Version 1 24/01/17


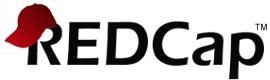
 [www.project-redcap.org](http://www.project-redcap.org/)

## Confidential

*Page 2 of 5*

### SECTION 2: Use of Printed Screening Referral

- 1. Did you take the printed referral the pharmacist gave you to your doctor's appointment?

... Is there a reason why you did not take the referral with you?

- 1. Did the doctor already have a copy of the referral form?
  2. Did the doctor take account of the information on the referral form during the appointment?

Yes No

Yes No

Unsure

Yes No

Unsure

### SECTION 3: Any Further Testing?

- 1. Did the doctor do any further tests to check for diabetes?

... Do you know why no further testing was done?

... If you are unsure, can you provide more information?

- 1. Do you know which tests they were? (please tick all that apply)

... Can you provide more details on the tests your doctor ordered?

- 1. Where were the tests conducted?

Yes No

Unsure

Fasting blood sugar

Oral Glucose Tolerance Test (OGTT) HbA1c

Other

Not sure which tests they were

Your Doctor's Surgery

A Pathology Collection Centre Other

... Can you provide the name(s) of the place(s) where the testing was done?

... Can you provide the address(es) of where the testing was done?

### SECTION 4: Have you been diagnosed with Diabetes or Prediabetes?

- 1. Since your pharmacy screening appointment, has a doctor diagnosed you with Prediabetes?
  2. Since your pharmacy screening appointment, has a doctor diagnosed you with Diabetes?

Yes No

Waiting for test results Unsure

Yes No

Waiting for test results Unsure

## Confidential

*Page 3 of 5*

### SECTION 5: Health Information and Advice

- 1. How did you feel about the way your pharmacist explained your screening test results?
  2. Were you given advice about HEALTHY EATING during your pharmacy appointment?

... How would you rate your pharmacist's advice about HEALTHY EATING?

... How would you rate the HEALTHY EATING brochure?

... Can you provide further details?

- 1. Were you given advice about EXERCISE during your pharmacy appointment?

... How would you rate your pharmacist's advice about EXERCISE?

... How would you rate the EXERCISE brochure?

... Can you provide further details?

- 1. Generally, how do you feel about the health information and advice you received from your pharmacist?

Very satisfied Satisfied

Neither satisfied or dissatisfied Dissatisfied

Very dissatisfied

Yes the pharmacist discussed it with me and gave me a brochure

Yes the pharmacist discussed it with me but I did not get a brochure

Yes the pharmacist gave me a brochure but did not discuss it with me

No I did not get any information Other

Very hepful Helpful

No opinion

Not very helpful Very helpful

Very hepful Helpful

No opinion

Not very helpful Very helpful

Yes the pharmacist discussed it with me and gave me a brochure

Yes the pharmacist discussed it with me but I did not get a brochure

Yes the pharmacist gave me a brochure but did not discuss it with me

No I did not get any information Other

Very hepful Helpful

No opinion

Not very helpful Very helpful

Very hepful Helpful

No opinion

Not very helpful Very helpful

Very satisfied Satisfied

Neither satisfied or dissatisfied Dissatisfied

Very dissatisfied

#
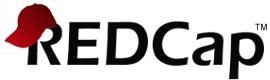
Appendix 20: Version 1 24/01/17

## Confidential

*Page 4 of 5*

### SECTION 6: Lifestyle Programs

- 1. Were you recommended to join a lifestyle program? (e.g. COACH, LIFE, GET HEALTHY)
  2. Did you join a lifestyle program?
  3. Which lifestyle program(s) did you join? (please tick all that apply)

Yes, by the pharmacist Yes, by the doctor

Yes, by both No

Yes No

LIFE

GET HEALTHY COACH

Other

... How would you rate the LIFE program? Not helpful at

all Very helpful

... How would you rate the GET HEALTHY program? Not helpful at

*(Place a mark on the scale above)*

all Very helpful

... How would you rate the COACH program? Not helpful at

*(Place a mark on the scale above)*

all Very helpful

... Which 'OTHER' lifestyle program did you join?

*(Place a mark on the scale above)*

... How would you rate this 'OTHER' lifestyle program? Not helpful at

all Very helpful

*(Place a mark on the scale above)*

... Is there a reason why you did not join a I plan to join later

lifestyle program as recommended? It was difficult to join

I did not think it was necessary to join Other

... Can you provide more details on why you did not join a lifestyle program?

### SECTION 7: Lifestyle Changes

- 1. Have you made any healthy lifestyle changes since attending the pharmacy diabetes screening service?
  2. Which of the following lifestyle changes have you made? (please tick all that apply)

... How did you change your cigarette (or other tobacco product) smoking habit? (please tick all that apply)

... How often do you eat vegetables or fruit?

Yes No

I changed my cigarette (or other tobacco) smoking habit

I increased my fruit and vegetable intake I increased my physical activity

I decreased my waist circumference

I made other healthy lifestyle changes

I quit smoking

I attempted to quit smoking

I reduced the amount I smoke per day

I increased the amount I smoke per day

Every day Not every day

## Confidential

*Page 5 of 5*

... If not every day, how often do you eat vegetables or fruit?

... On average, would you say you do at least 2.5 Yes

hours of physical activity per week (for example, 30 No minutes a day on five or more days a week)?

... If not at least 2.5 hours per week, how often do you exercise?

... If you've decreased your waist circumference, do you know by how much? If so, record it here (in cm).

... Can you provide further details about the other healthy lifestyle changes you made?

### SECTION 8: Satisfaction with Pharmacy Diabetes Screening Service

- 1. How would you describe the pharmacy diabetes screening service?
  2. Generally, how do you feel about the pharmacy diabetes screening service?

Very professional Professional

No opinion Unprofessional Very unprofessional

Very satisfied Satisfied

Neither satisfied or dissatisfied Dissatisfied

Very dissatisfied

... Can you tell us why you were satisfied with the pharmacy diabetes screening service?

... Can you tell us why you were dissatisfied with the pharmacy diabetes screening service?

- 1. What is your opinion about the diabetes screening Strongly support service being available in your pharmacy in the Support

future? No opinion

Do not support Strongly do not support

... Can you tell us why you support the pharmacy diabetes screening service?

... Can you tell us why you do not suppport the pharmacy diabetes screening service?

- 1. Would you recommend the pharmacy diabetes Yes

screening service to a friend or family member? No

- 1. Are there any other comments you wish to make regarding the diabetes screening service?

**APPENDIX 20**

**Version 1: 24/10/17**
