# Supplementary material for: Participant and GP perspectives and experiences of screening for undiagnosed type 2 diabetes in community pharmacy during the Pharmacy Diabetes Screening Trial
Source: BMC Health Serv Res. 2023 Dec 1;23:1337. doi: 10.1186/s12913-023-10269-1 (PMC10693079; doi:10.1186/s12913-023-10269-1)
Supplement: Supplementary file 2 — Additional file 2. [file 12913_2023_10269_MOESM2_ESM.docx]

**Additional file 2**

**General Practitioner Evaluation Survey**

**Pharmacy Diabetes Screening Trial**

**EVALUATION SURVEY FOR GENERAL PRACTITIONERS**

Dear Doctor…….

You are being contacted because one or more of your patients recently took part in this screening project undertaken in community pharmacy. You should have received a report from the patient’s pharmacy, and possibly other correspondence about the trial more generally.

We are currently undertaking a complete evaluation of this project and an important part of this includes an understanding of the general practice perspective. Development of this project was heavily influenced by the need to ensure that pharmacy screening programs are integrated with general practice to ensure appropriate medical assessment and follow up after screening. We are very keen to understand the impact you felt it had both on you and your patient(s).

This survey will only take 5-10minutes. Are you happy to proceed?

I will read out the questions relating to the **Pharmacy Diabetes ScreeningTrial**. Your honest feedback is important to evaluate the program.

Please indicate your extent of agreement or disagreement with the following statements where “1” equals strongly disagree “2” disagree, 3 “neither agree nor disagree”, “4” agree and “5” strongly agree.

| 1. | **Strongly**  **Disagree** | **Disagree** | **Neither Agree or Disagree** | **Agree** | **Strongly Agree** | **Unsure** |  |  |  |  |
| --- | --- | --- | --- | --- | --- | --- | --- | --- | --- | --- |
|  | | | | | |  |  |  |  |  |
| 1. The pharmacy diabetes screening was a valuable service for my patient(s). | **1** | **2** | **3** | **4** | **5** | **9** |  |  |  |  |
| 2. My patient(s) showed more interest in their health as a result | **1** | **2** | **3** | **4** | **5** | **9** |  |  |  |  |
| 3. I felt fully informed about what happened with my patients during the diabetes screening | **1** | **2** | **3** | **4** | **5** | **9** |  |  |  |  |
| 4. The screening referral worked well to inform me about what happened with my patients during the diabetes screening | **1** | **2** | **3** | **4** | **5** | **9** |  |  |  |  |
| 5. The pharmacist(s) made reasonable efforts to respond to any requests I made | **1** | **2** | **3** | **4** | **5** | **9** |  |  |  |  |
| 6. Tests and measurements  undertaken by pharmacist(s) were performed correctly |  | **2** | **3** | **4** | **5** | **9** |  |  |  |  |
| 7. Advice provided to patients after screening always seemed reasonable | **1** | **2** | **3** | **4** | **5** | **9** |  |  |  |  |
| 8. Recommendations for follow-up care made by the pharmacist(s) were appropriate | **1** | **2** | **3** | **4** | **5** | **9** |  |  |  |  |
| 9. The pharmacy screening led me to conduct extra investigations for diabetes fro referred patients | **1** | **2** | **3** | **4** | **5** | **9** |  |  |  |  |
| 10. The Pharmacy Diabetes Screening program led me to conduct extra investigations for my other patients | **1** | **2** | **3** | **4** | **5** | **9** |  |  |  |  |
| 11. Overall, I trust the pharmacy (ies) involved to deliver a competent diabetes screening service | **1** | **2** | **3** | **4** | **5** | **9** |  |  |  |  |
| 12. The Pharmacy Diabetes Screening Program intefered with my relationship with my patient | **1** | **2** | **3** | **4** | **5** | **9** |  |  |  |  |

13. On a scale of “1” to “7” How would you descibe the community pharmacy(ies) as a place to conduct this type of diabetes screening program?

*Highly unsuitable* *1………2 ….3 …..4……..5……....6……..7* *Highly suitable*

Would you like to give a reason for your rating?

………………………………………………………………………………………………………………………………………………………………………………………………………

12. Did you experience any of the barriers and challenges listed below? *(tick as many as apply))*

❑ This service was too intrusive on my current practice.

❑ I found this type of service too time consuming.

❑ Some of the advice provided by pharmacists conflicted with my advice

❑ I disagree with the need for screening of some or all of the patients screened

13. What, if anything, did you **like** **most** about this Pharmacy Diabetes Screening Program?

…………………………………………………………………………………………………………

………………………………………………………………………………………………………………………………

14. What, if anything, did you **dislike** about this Pharmacy Diabetes Screening Program?

…………………………………………………………………………………………………………

………………………………………………………………………………………………………………………………

14. How could your experience with this pharmacy diabetes screening have been improved?

……………………………………………………………………………………………………………………………………

…………………………………………………………………………………………………………………………………

Please provide any other comments you think are relevant:

……………………………………………………………………………………………………………………………………

…………………………………………………………………………………………………………………………………

…………………………………………………………………………………………………………………………………

…………………………………………………………………………………………………………………………………

…………………………………………………………………………………………………………………………………

…………………………………………………………………………………………………………………………………

…………………………………………………………………………………………………………………………………

…………………………………………………………………………………………………………………………………

**There are no further questions. Thank you for your participation and support.**
